# RNA-Seq analyses reveal the order of tRNA processing events and the maturation of C/D box and CRISPR RNAs in the hyperthermophile *Methanopyrus kandleri*

**DOI:** 10.1093/nar/gkt317

**Published:** 2013-04-25

**Authors:** Andreas A. H. Su, Vanessa Tripp, Lennart Randau

**Affiliations:** ^1^Max-Planck-Institute for Terrestrial Microbiology, Max Planck Research Group: Prokaryotic Small RNA Biology, Karl-von-Frisch Strasse 10, 35037 Marburg, Germany and ^2^LOEWE Center for Synthetic Microbiology (Synmikro), 35037 Marburg, Germany

## Abstract

The methanogenic archaeon *Methanopyrus kandleri* grows near the upper temperature limit for life. Genome analyses revealed strategies to adapt to these harsh conditions and elucidated a unique transfer RNA (tRNA) C-to-U editing mechanism at base 8 for 30 different tRNA species. Here, RNA-Seq deep sequencing methodology was combined with computational analyses to characterize the small RNome of this hyperthermophilic organism and to obtain insights into the RNA metabolism at extreme temperatures. A large number of 132 small RNAs were identified that guide RNA modifications, which are expected to stabilize structured RNA molecules. The C/D box guide RNAs were shown to exist as circular RNA molecules. In addition, clustered regularly interspaced short palindromic repeats RNA processing and potential regulatory RNAs were identified. Finally, the identification of tRNA precursors before and after the unique C8-to-U8 editing activity enabled the determination of the order of tRNA processing events with termini truncation preceding intron removal. This order of tRNA maturation follows the compartmentalized tRNA processing order found in Eukaryotes and suggests its conservation during evolution.

## INTRODUCTION

Organisms that belong to the domain Archaea are often adjusted to the harshest environments present on earth. *Methanopyrus kandleri* is a methanogenic archaeon that can survive extreme heat and pressure conditions. One of the earliest archaeal genome sequences was obtained for *M. kandleri* AV19 (1). This organism was isolated near hydrothermal vents from the Gulf of California at a depth of 2000 m below sea level and at temperatures of 84–110°C (2). More recently, *M. kandleri* strain 116 was isolated and shown to proliferate at 20 MPa pressure and 122°C (3), which extended the known upper temperature limit for life. The search for features that allow growth at these temperatures revealed that *M. kandleri* sustains high intracellular concentrations of the trivalent anion cyclic 2,3-diphosphoglycerate, and enzymes isolated from this organism can require over 1 molar salt concentrations to ensure their stability and activity (4,5).

A large number of orphan genes were identified in the *M. kandleri* genome, which revealed several unusual enzymes including the topoisomerase V and a two-subunit reverse gyrase (6,7). One orphan gene encodes a unique cytidine deaminase that acts on transfer RNA (tRNA) base 8 (CDAT8) (8). All organisms from all three domains of life contain tRNA genes with a conserved T residue at position 8 and the folding of tRNA molecules involves tertiary interactions between U8 and the equally conserved base A14. The only known exception to this rule is the minimal set of 34 tRNA genes that is present in the *M. kandleri* genome and that contains 30 genes that have an unusual C base at position 8. The tRNA precursors that contain a C8 base are then edited by CDAT8 deamination to ensure that mature tRNA molecules contain the universal U8 required for proper folding.

It has been suggested that the acquisition of the orphan genes was mediated by viruses (9) and that these rare genes evolved in the viral community. The identification of five anti-viral clustered regularly interspaced short palindromic repeats (CRISPR) systems in the genome of *M. kandleri* underlines the importance of virus—host interactions in extreme environments.

The phylogenetic positioning of *M. kandleri* among other Archaea is debated. *M. kandleri* is the only known member of the class Methanopyrales belonging to the phylum Euryarchaeota. The 16S ribosomal RNA (rRNA) gene-based phylogenetic studies placed the *M. kandleri* branch deep at the root of the archaeal tree (10), whereas whole genome trees include *M. kandleri* with other methanogens in a monophyletic group (11). Although the latter scenario is supported by the observation of accelerated evolution of transcriptional proteins, *M. kandleri* is still considered to represent an organism that is phylogenetically close to the Last Universal Common Ancestor at the root of the tree of life (12).

The extreme growth conditions of *M. kandleri*, the divergent phylogenetic positioning and the unique tRNA editing events raise question about the RNA properties of this organism. In the present study, we applied RNA-Seq deep sequencing methodology to isolated enriched small RNA samples from *M. kandleri* to obtain an insight into the small RNA metabolism at extreme temperatures and to follow tRNA C-to-U editing activity. A small RNome (sRNome) set was characterized that highlights the importance of the modification of structured RNAs and the defence against viruses. Sequencing of tRNAs with and without C8 editing revealed tRNA processing intermediates and highlighted that termini truncation precedes intron removal.

## MATERIALS AND METHODS

### Cell cultivation and RNA isolation

*Methanopyrus kandleri* cells were a kind gift of D. Söll. The organism was grown in the Archaeenzentrum Regensburg (H. Huber, M. Thomm, K. Stetter) in a 300 l fermenter as described (1). Total RNA was isolated by SDS-lysis of the cell pellet and phenol/chloroform extraction, and small RNAs were purified from total RNA using the MirVana RNA extraction kit (Ambion).

### RNA-*s*equencing

Six different *M. kandleri* small RNA libraries were prepared for sequencing. The following RNA pre-treatment protocols were applied before adapter ligation: Sample 1: 10 µg of *M. kandleri* sRNA was incubated in 1× T4 polynucleotidekinase (T4 PNK) buffer (NEB) for 6 h at 36°C in a total volume of 50 µl with no additional treatment. Sample 2: 10 µg of *M. kandleri* sRNA was incubated in a buffer containing 50 mM sodium phosphate, 1 mM EDTA and 0.36% H_2_O_2_ at 20°C for 3 h to facilitate dethiolation of RNA. Sample 3: 10 µg of *M. kandleri* sRNA was incubated in a buffer containing 170 mM Tris–HCl (pH 8.8) at 37°C for 3 h to facilitate deacetylation of tRNA molecules. Samples 1–3 resulted in highly similar RNA-Seq sequencing output. Samples 4–6 were treated with T4 PNK to ensure proper termini for adapter ligation to RNA molecules that contain either 5′ OH termini or 3′ phosphate termini. Sample 4: 10 μg of small RNA was incubated at 37°C for 6 h with 20 U T4 PNK and in 1× T4 PNK buffer in a total volume of 50 μl. Subsequently, 2 mM adenosine triphosphate (ATP) and 10 U T4 PNK were added, and the reaction mixture was incubated for 1 h at 37°C to generate monophosphorylated 5′ termini. Sample 5: The protocol for Sample 4 was followed but the final addition of 2 mM ATP was omitted. Sample 6: The protocol for Sample 4 was followed, but 2 mM ATP was added from the beginning. Samples 4 to 6 resulted in highly similar RNA-Seq sequencing output. RNA libraries were prepared with an Illumina TruSeq RNA Sample Prep Kit (Ambion), and sequencing on an Illumina HiSeq2000 sequencer was performed at the Max-Planck Genomecentre, Cologne (Max Planck Institute for Plant Breeding Research, Köln, Germany).

### Identification of small RNA species

Sequencing reads were trimmed by (i) removal of Illumina TruSeq linkers and poly-A tails and (ii) removal of sequences using a quality score limit of 0.05. A total of 83 338 855 reads with an average length of 61 nt were mapped to the *M. kandleri* reference genome (GenBank: NC_003551) with CLC Genomics Workbench 5.5 (CLC Bio, Aarhus, Denmark). The following mapping parameters were used: mismatch cost, 2; insertion cost, 3; deletion cost, 3; length fraction, 0.5; similarity, 0.8. This program was also used to determine the coverage of individual RNA molecules. All predicted RNA molecules and their termini were manually verified, and all intergenic regions were checked for the presence of RNA molecules with coverage of >500 reads. Target prediction of identified C/D box sRNAs was performed with the PLEXY tool using the default parameters (13). Provided possible target RNA sequences were as follows: (i) all rRNAs and tRNAs sequences and (ii) all identified sRNAs (Supplementary Table S1). The following algorithms were used for further computational analysis of the data: RNA folding [Mfold (14)], tRNA gene prediction [tRNAScan-SE (15), genomic tRNA database (16)], small nucleolar RNA (snoRNA) gene prediction [snoscan (17)], crRNA identification [crisprdb (18)], RNA alignments [ClustalW2 (19)] and RNA visualization [VARNA (20)]. Gene annotations were obtained from GenBank.

### Inverse *Reverse transcriptase*-*polymerase chain reaction*

In all, 10 ng of an *M. kandleri* sRNA preparation were treated with Superscript III reverse transcriptase (Invitrogen) and primers against C/D box sRNAs 5, 11, 16, 17, 27 to generate complementary DNA (cDNA) for the detection of circular sRNA molecules. The RNA was denatured at 100°C for 5 min and cooled on ice for 5 min to facilitate reverse transcription at 55°C for 30 min. Inactivation of the reverse transcriptase was carried out at 70°C for 15 min. Subsequently, the cDNA products were polymerase chain reaction (PCR) amplified with Platinum Taq DNA polymerase (Invitrogen) using forward and reverse primers. The following primers were used:

C/D_5_For: 5′-GATCCCATCCTCATCCCAC-3′,

C/D_5_Rev: 5′-GATCTGGGAGGCCGTTAC-3′,

C/D_11_For: 5′-GCGTGGGGTAGCATCGTC-3′,

C/D_11_Rev: 5′-ACCCCGATGAGGAGGAAC-3′,

C/D_16_For: 5′-GTTCGTCGGCCTACCTCG-3′,

C/D_16_Rev: 5′-GGGATGACGACCCCTGG-3′,

C/D_17_For: 5′-CGATCCTGCGACCACTCC-3′,

C/D_17_Rev: 5′-GCGGTTGTTCGCTTCTTCATC-3′,

C/D_27_For: 5′-GCTCAATCTTCATCCACAGGATC-3′,

C/D_27_Rev: 5′-AGCCCGGCACTGACTCG-3′.

The PCR amplificates were cloned into a pCR2.1 TOPO vector (Invitrogen) and sequenced (Eurofins MWG Operon).

### Data availability

The RNA-Seq data are available at NCBI’s Gene Expression Omnibus website as series GSE44979.

## RESULTS

### The small RNA profile of *Methanopyrus kandleri*

**Figure 1.**
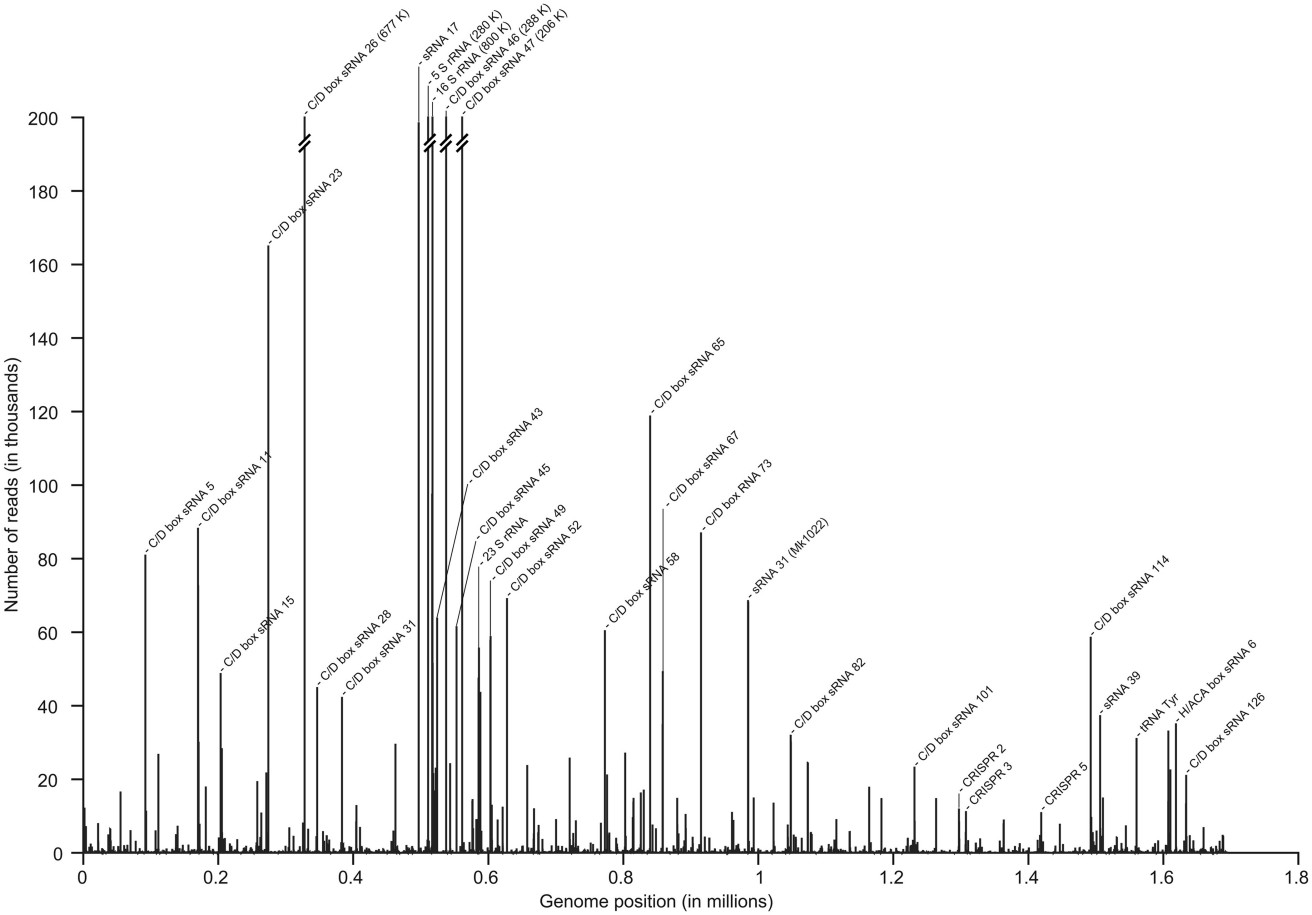
The sRNome of *M. kandleri*. The overview graph illustrates the genome-wide coverage of Illumina HiSeq2000 reads mapped to the *M. kandleri* AV19 genome (Genbank: NC_003551, 1694969 bp). Prominent peaks were analyzed at single-nucleotide resolution, and abundant sRNAs (e.g. C/D box sRNAs) were identified. For peaks that cover over 200 000 reads, the number of reads is given in brackets.

RNA-Seq methodology was used to facilitate the genome-wide analysis of small RNA production at single nucleotide resolution in *M. kandleri*. RNA molecules were isolated from *M. kandleri* cells, and small RNA molecules (<200 nt) were selectively enriched. The RNA preparation was split into six fractions and subjected to different RNA modification procedures (detailed in the ‘Materials and Methods’ section) before adapter ligation. Three RNA samples were treated with T4 PNK to enable sequencing of RNAs with 5′ OH termini. Six independent library preparations were used for HiSeq2000 RNA-Seq sequencing. The obtained reads were mapped to the 1.69 bp long *M. kandleri* genome. In total, these mappings contain 83 338 855 reads with an average length of 61 nt. Clear differences for the obtained sRNome coverage were observed for the three RNA samples that were treated with T4 PNK in comparison with the three samples without this treatment. This allowed us to focus our small RNA analysis on two of these conditions and provided us with four further mappings that we used for the assessment of the reproducibility of our observations. The overview of the genome-wide RNA profile of *M. kandleri* reveals that most sequence reads were obtained for fragments of the 5S and 16S rRNAs as well as for small C/D box sRNAs (Figure 1). Members of this class of RNA modifying guide RNAs are abundant in *M. kandleri* and will be discussed in detail later in the text. In addition, several potential regulatory sRNAs were identified. Finally, mature crRNA were selectively enriched in the RNA samples treated with T4 PNK.

### Abundance of tRNA molecules

Sequence reads that represent tRNAs are highly underrepresented in RNA-Seq studies. In the cell, tRNAs are usually found to represent the most abundant class of RNAs, which correlates with their high demand during protein biosynthesis. However, tRNA genes are often covered only by a few hundred reads in our RNA-Seq mappings. In addition, these sequence reads often span only a fraction of the tRNA genes. It has been noted before that the high level of tRNA modification and the stable secondary and tertiary structure of these molecules poses difficulties for the reverse transcriptase during the cDNA preparation step for RNA-Seq libraries. This is especially true for the tRNAs of *M. kandleri* that have to function at growth temperatures ∼100°C, which is ensured by tRNA stems that nearly exclusively are formed by G-C base pairing and by an exceptionally diverse population of modified nucleosides (21). On the other hand, the obtained fragmented tRNA sequence reads offer insights into the maturation and processing stages as, for example, the insertion of bulky modifications can be pinpointed by reverse transcriptase stalling (22). The tRNA genes with a significant number of mapped reads are tRNA^Tyr^ (31353 reads) and tRNA^Asp^ (23871 reads). Interestingly, in both cases, the majority of the reads represent tRNA precursors that still contain a 5′ leader sequence (5′-GAGGGGTGCGGGA-3′ for tRNA^Tyr^ and 5′-GGAGGGATGAGA-3′ for tRNA^Asp^). These precursors most likely do not contain modifications that could block reverse transcription. The tRNA^Tyr^ species is the only tRNA that is required to start with a C as the first base to ensure proper aminoacylation by the archaeal tyrosyl-tRNA synthetase (23). As transcription initiation prefers purine residues, RNase P activity is required to mature the 5′ end of functional tRNA^Tyr^. Most *M. kandleri* tRNAs contain short 5′-terminal leader sequences that contain multiple G residues commonly found at archaeal transcription initiation sites (Supplementary Table S1). Three tRNA genes displayed problematic annotations. A tRNA^Leu^ isoacceptor lacked three 5′-terminal residues, and the two tRNA^Glu^ isoacceptors display an unusual second intron in the tRNAs’ D-loop, which was previously predicted to be recognized by the splicing endonuclease (24). No sequence reads for tRNA^His^ species were observed that contain a G-1 residue. This hallmark of tRNA^His^ species is posttranscriptionally added by a special enzyme, the tRNA^His^ guanylyltransferase, which is also present in *M. kandleri*. It is possible that this reaction happens late during tRNA^His^ maturation after the incorporation of modifications that abolishes reverse transcription efficiency or that the G-1 is further modified in a fashion that prevents 5′-terminal adapter ligation.

### Maturation of tRNA molecules

**Figure 2.**
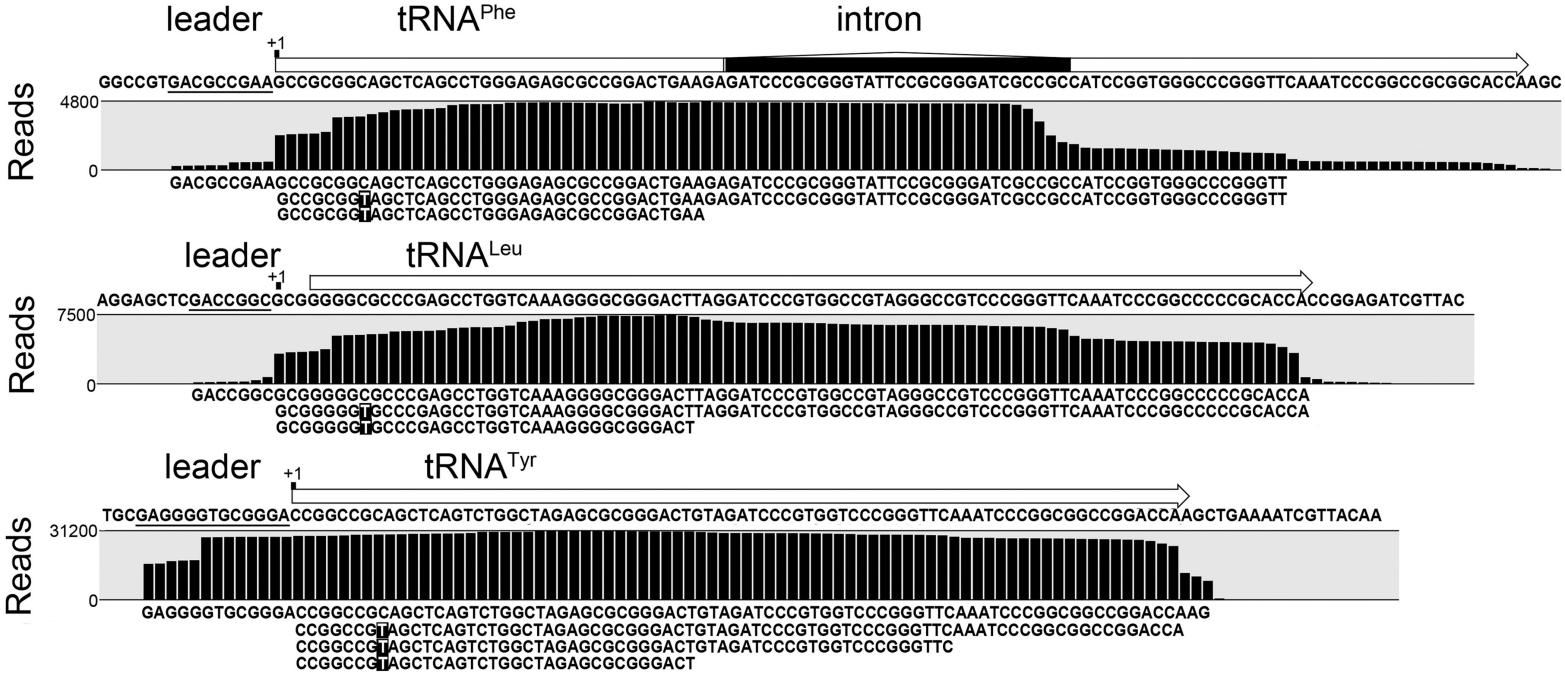
Detection of tRNA processing events. Indicated is the coverage of Illumina Hiseq2000 reads at single nucleotide resolution for three exemplary tRNA genes. The Genbank annotation of the three tRNA genes and an intronic sequence is depicted above the coverage graphs and the identified mature 5′ termini are indicated by ‘+1’. The 5′ end of tRNA^Leu^ was misannotated. Representative reads that allow the determination of the tRNA precursor processing state are given below the coverage graphs: (i) tRNAs with 5′-terminal leader sequences before RNase P processing, (ii) tRNA precursors with intronic sequences, (iii) tRNAs with C8-to-U8 editing (highlighted in black) and (iv) shortened tRNA reads that suggest the presence of tRNA modifications.

**Figure 3.**
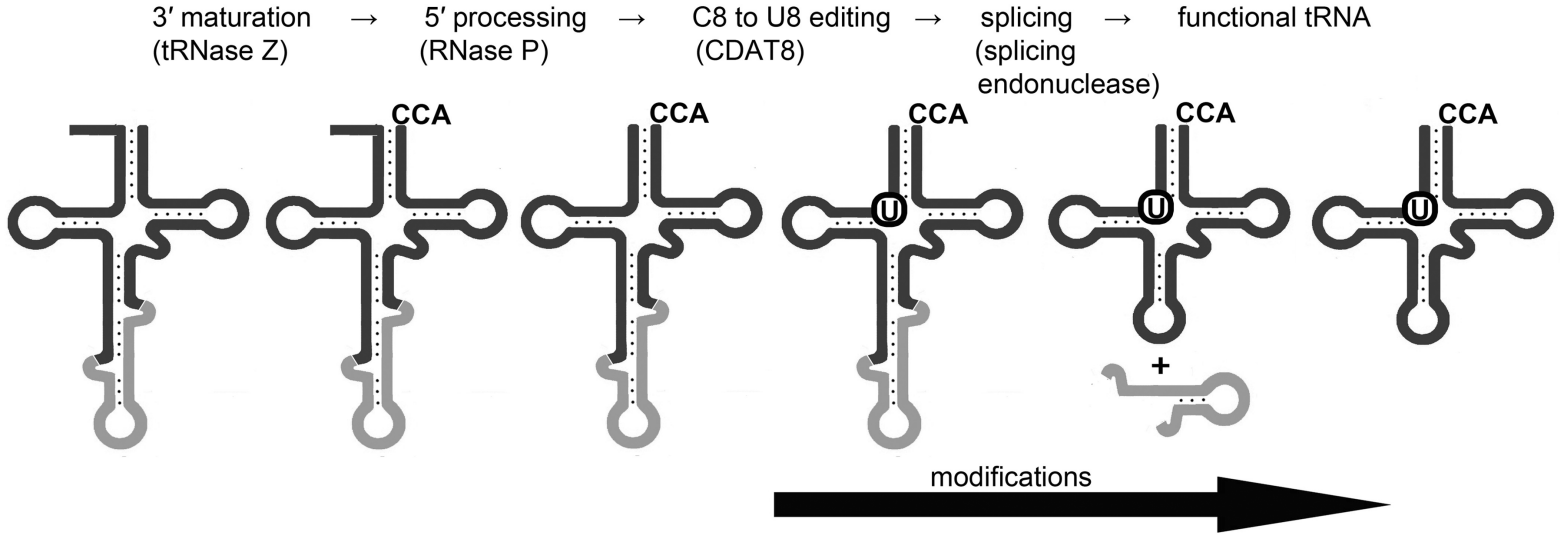
Deduced order of tRNA processing events. Sequencing reads that represent different tRNA processing states were quantified (Supplementary Table S1) and indicate the presented order of tRNA processing events.

Reads that mapped to the regions of tRNA genes were used to distinguish between different stages of tRNA processing. The following different tRNA maturation intermediates were identified: (i) tRNAs with 5′-terminal leader sequences, (ii) tRNA with intronic sequences, (iii) tRNAs with C8 to U8 editing by CDAT8 and (iv) tRNA fragments (possibly caused by bulky modifications). The first three intermediates can be inferred from sequencing reads at single nucleotide resolution that were mapped to the tRNA genes in the reference genome or tRNA genes in a modified reference genome that contained the C8–T8 exchange. The different precursors were quantified (Supplementary Table S1). The occurrence of different tRNA maturation intermediates that did or did not contain features of other intermediates allowed for the generation of an order of tRNA processing events. Here, the C8–U8 editing serves as an ideal marker for one clearly distinguishable modification event. Ninety-nine percent of all tRNAs that contained the edited mature U8 base were identified by reads that started with the +1 base (Supplementary Table S1 and Figure 2). Only tRNA precursors that still contain C8 or the four tRNAs that naturally have the U8 base are commonly found with 5′-terminal leader sequences. Thus, it can be deduced that 5′ tRNA processing by RNase P precedes tRNA editing. In contrast, 89% of sequence reads for tRNAs with introns still contain this intron and also exhibit the C8–U8 exchange. In addition, these tRNAs often terminate prematurely at positions with presumed modifications (Supplementary Table S1 and Figure 2). This indicates that certain modifications are inserted into the tRNA molecule before CDAT8 activity, and that the removal of tRNA introns occurs at the later stages of tRNA maturation. In addition, the two tRNA^Glu^ isoacceptors are represented by reads with C8 to U8 editing (114 and 294 reads, respectively) that still contain the unusual intron in the D-loop of the tRNA. This indicates that, although CDAT8 requires a matured acceptor stem, it tolerates disruption and insertion of structured sequences in the D-loop and the anticodon-loop. This observation is in agreement with a model of CDAT8 interaction with the T-stem/loop and acceptor-stem portion of 30 different tRNA substrates (8). Removal of the D-loop intron appears to trail the removal of the canonical intron. In conclusion, the order of observed tRNA processing events is as follows: (i) 3′ processing, (ii) 5′ processing, (iii) modifications, C-to-U editing, (iv) intron removal (Figure 3).

### Identification of C/D box and H/ACA box sRNAs

Most sequencing reads were obtained for C/D box sRNAs. These 55–97 nt small RNA molecules use a guide sequence located between the conserved C and D box RNA motifs to target RNA (most commonly rRNAs) for 2′-*O*-methylation (25). A different and less abundant class of guide RNAs, the H/ACA box sRNAs direct RNA pseudouridylation. A total of 126 C/D box sRNAs and 6 H/ACA box sRNAs were found in *M. kandleri* (Supplementary Table S1). The abundance of individual C/D box sRNAs was found to be highly variable in the cell, despite conserved length, structure and functions. Some C/D box sRNAs were covered by few hundred reads, whereas others were among the most abundant sRNA molecules in the cell covered by hundred thousands of sequencing reads (Figure 1 and Supplementary Table S1). We identified sequence coverage and manually annotated termini and potential C and D boxes for all detected C/D box sRNAs (Supplementary Table S1). Computational analyses using the algorithm ‘snoscan’ (17) identified 97 potential C/D box sRNAs of which 79 were verified experimentally and 17 potential C/D box sRNAs that were classified as ‘questionable’ of which only two were verified. Forty-five C/D box sRNAs were not predicted computationally. All predictions of C/D box sRNAs within protein-coding genes were found to be erroneous. Potential targets for the guide sequence of these small RNAs were computationally predicted (Supplementary Table S1). These targets include not only rRNAs but also tRNAs and other non-coding RNAs. Interestingly, the target with the overall best score is the 5′ leader region of a newly identified H/ACA box sRNA (Supplementary Table S1 and Supplementary Figure S1), which suggests potential cooperative activity of guide RNAs required for different RNA modifications. We observed the highest number of sequence reads that map to a protein-coding gene for the gene MK0859 coding for GAR1, a member of the H/ACA small ribonucleoprotein complex. Analysis of the GAR1 mRNA sequence revealed a highly stable 3′-terminal hairpin structure with 10 consecutive G-C base pairs within the open reading frame that might influence mRNA stability (Supplementary Figure S2).

**Figure 4.**
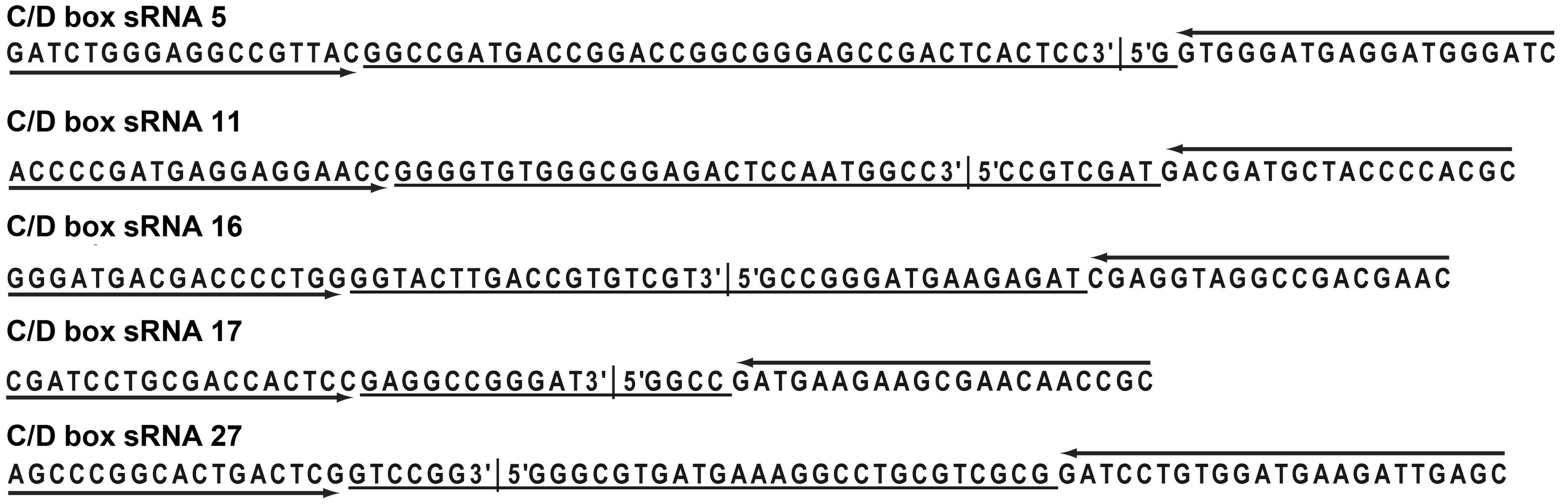
Inverse RT-PCR verifies circular C/D box sRNA formation. One hundred and twenty-six C/D box sRNAs were identified and permuted sequencing reads suggested circular RNA molecules (Supplementary Table S1). Selected circular C/D box sRNA molecules were amplified by inverse RT-PCR with outward facing primers (arrows) and amplificates were sequenced. Sequencing information between primer sequences is underlined and circularization junctions are marked.

Permuted sequencing reads indicated that most C/D box sRNA exist as circular RNA molecules in the cell. In some cases, the circularized form was found to be more common than the linear RNA molecule (Supplementary Table S1). Circular RNA molecules require internal cleavage to facilitate adapter ligation to the permuted RNA, which suggests that such circular molecules are likely underrepresented in RNA-Seq studies. To verify that circularization is not an artifact occurring during RNA library preparation, we performed inverse reverse transcriptase-polymerase chain reaction (RT-PCR) amplification with selected C/D box sRNA candidates. The amplification products were sequenced and confirmed the presence of the circularization sites (Figure 4).

### crRNAs

**Figure 5.**
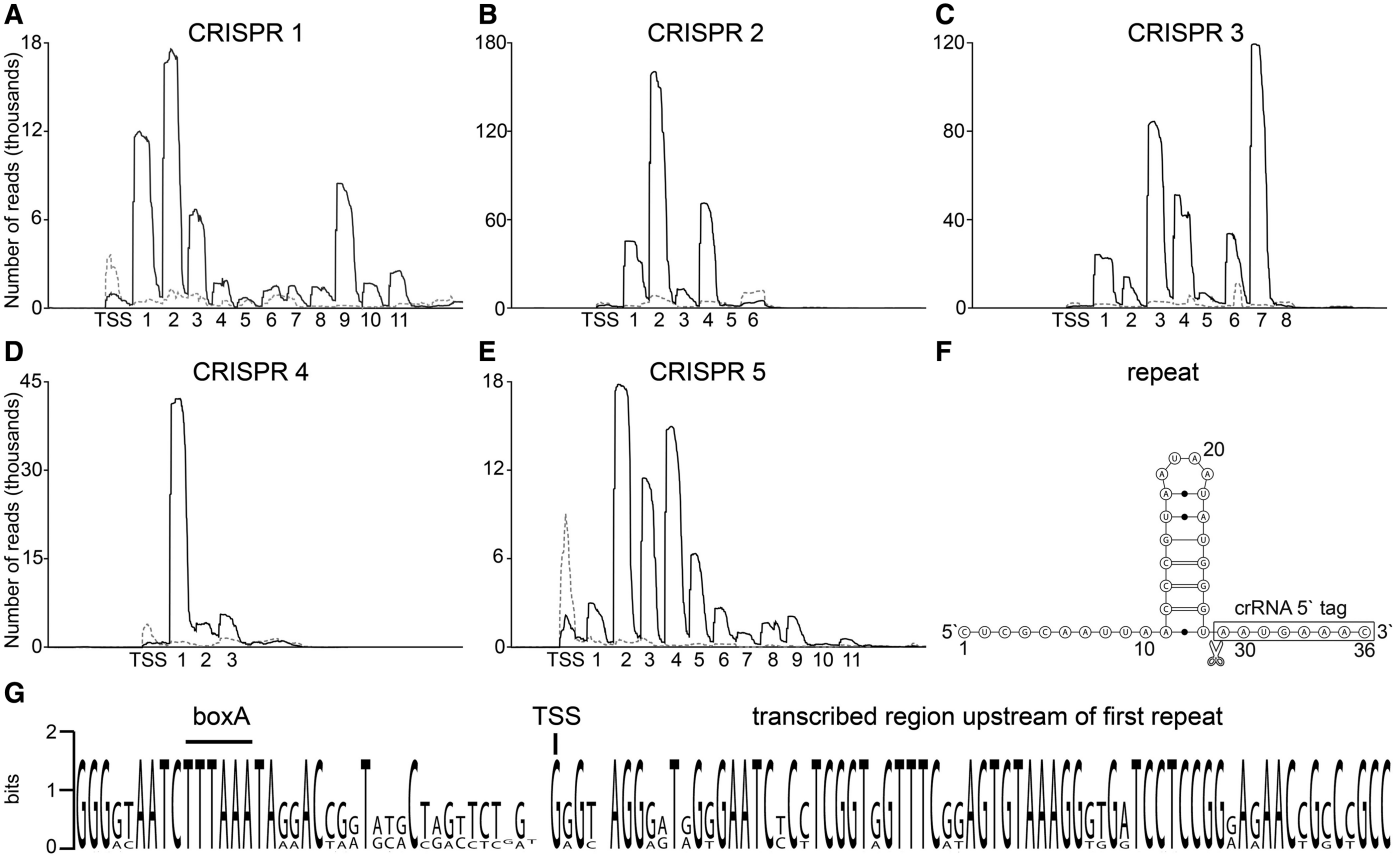
Detection of crRNA abundance and processing. (**A–E**) Illumina Hiseq2000 reads of T4 PNK-treated (black line) and untreated (gray dashed line) RNA samples were mapped to CRISPR loci 1–5 in the *M. kandleri* genome. The number of identified processed crRNAs and the detected transcription start site (TSS) are indicated. (**F**) Shown is the consensus repeat sequence and proposed structure. The detected cleavage site is indicated, and mature crRNAs contain an 8 nt 5′ tag. (**G**) A sequence logo of the aligned regions upstream of the first repeat in CRISPR loci 1–5 highlights a conserved transcription start site (TSS) and a boxA promoter sequence.

The genome of *M. kandleri* harbors five CRISPR clusters. The characteristic CRISPR repeat sequences are interspaced by sequences that can be derived from viral DNA (so-called spacers) and are relics of viral infections. Our RNA-Seq analyses revealed that all five CRISPR clusters are active, i.e. they are transcribed and processed into small crRNAs that have the potential to mediate immunity against viruses that are recognized via base complementarity between viral DNA and crRNA spacer sequence. Based on the presence of different sets of CRISPR-associated (Cas) genes and different repeat sequences and structures, CRISPR/Cas subtypes have been classified (26). *Methanopyrus kandleri* contains two sets of Cas proteins that belong to subtype III-A and III-B, respectively, and an Argonaute gene located between genes encoding Cas1 and Cas2. Mature crRNAs of other CRISPR subtypes were shown to harbor 5′- hydroxyl- and potential 2′, 3′- cyclic phosphate ends (27). These termini are incompatible with the adaptor ligation needed for RNA sequencing. To produce suitable ends for RNA sequencing of mature crRNAs and to identify whether these termini are present in *M. kandleri* crRNAs, a set of three RNA preparations was treated with T4 PNK. This enzyme adds 5′ phosphates and removes potential 3′ phosphate groups from mature crRNAs before library preparation and RNA-Seq. It is evident that T4 PNK treatment is required for the detection of all crRNAs of the five CRISPR clusters in *M. kandleri* (Figure 5A–E and Supplementary Table S1). Most of the sequenced crRNAs harbor an 8 nt 5′ tag derived from cleavage within the repeat sequence, whereas the 3′ ends are gradually degraded and did not show a distinct cleavage site (Figure 5F). Our sequencing data revealed two crRNAs at the end of CRISPR cluster 4 and CRISPR cluster 5, respectively, which are not listed in the CRISPR database (18). These crRNAs are processed, even though the 3′-terminal repeat sequences show degeneration with several mutations. Sequencing of RNA samples without T4 PNK treatment revealed the transcription start site of the long CRISPR precursor RNA, whereas mature crRNAs were virtually absent. Mapping of the transcription start sites revealed that all five CRISPR precursors start with a G located 71–73 nt upstream of the first repeat and that the box A promoter element 5′-TTTAAA-3′ was conserved (Figure 5G and Supplementary Table S1).

### Regulatory small RNAs and other sRNA classes

After identification of rRNAs, tRNAs, C/D box sRNAs, H/ACA box sRNAs, SRP RNA, RNase P RNA and crRNAs, the *M. kandleri* sRNome revealed a number of sRNAs that did not belong to any of these RNA families (Supplementary Table S1). These RNAs include intergenic sRNAs and *cis*-antisense RNAs, which could provide means to regulate gene expression. An Lsm protein (MK0220) to facilitate interaction of regulatory sRNAs with their mRNA targets is present in *M. kandleri*. Unfortunately, the lack of genetic tools for *M. kandleri* does not permit function analyses for these sRNA candidates.

## DISCUSSION

*Methanopyrus kandleri* grows near the upper temperature limit for life. These extreme conditions provide challenges for the production and stability of all cellular macromolecules. Proper folding and the stable maintenance of the folded structure have to be guaranteed for structured non-coding RNA molecules. One strategy is the use of G-C base pairing over A-T base pairing in RNA structure elements (28). Additionally, posttranscriptional modifications play a crucial role in attenuating thermal RNA denaturation (29). Mass spectrometry analysis revealed an exceptionally diverse population of modified nucleosides in *M. kandleri* tRNAs (21) and a high degree of 2′-*O*-methylated nucleosides (30). These observations are in agreement with our detection of record numbers of C/D box sRNAs that are required to guide 2′-*O*-methylation of other RNA molecules and that underlines the importance of RNA modifications in hyperthermophilic organisms.

In addition, the stability of the guide RNAs that are involved in these RNA modifications needs to be guaranteed. C/D box sRNAs contain conserved RNA motifs (box C and box D sequences) that are required to form a kink-turn RNA secondary structure motif on binding of the L7Ae protein (31,32). Base pairing of the RNA termini ensures kink-turn formation in eukaryotic C/D box snoRNA (33). However, in *M. kandleri*, reverse complementary sequences located at the C/D box sRNA termini are either absent or too short to ensure stable RNA stems at the organism’s growth temperature (Supplementary Table S1). Therefore, the observed circularization of C/D box sRNA might be used to stabilize these important guide RNAs at elevated temperatures.

Circular C/D box sRNAs have been found in three different archaeal organisms that all share a hyperthermophilic lifestyle (34–36), which suggests that RNA circularization is a conserved feature in this extreme environment. RNA circularization was shown to increase RNA stability and to prevent nuclease degradation (37), and it has recently been suggested that circular RNAs are common in all three domains of life (38). However, the mechanism of archaeal C/D box sRNA circularization is currently still unknown.

A different RNA modification that is currently only known to occur in *M. kandleri* cells is cytidine deamination of tRNA at position 8, catalyzed by the orphan deaminase CDAT8. In all, 30 of 34 tRNA genes are transcribed as primary transcripts with a C at position 8 that is edited to U in the mature tRNA molecule. We took advantage of this unique editing marker to differentiate tRNA precursors in the RNA-Seq data and revealed that C-to-U editing occurs after termini truncation but before intron removal. This allows us to deduce the order of these processing events in a wild-type prokaryotic non-compartmentalized cell. In eukaryotic cells, compartmentalization can be used to order tRNA precursor maturation events. The tRNA genes are transcribed in the nucleus and tRNA termini are trimmed and matured. In yeast, these tRNAs are exported into the cytoplasm, where subsequently modifications are introduced and introns are spliced (39). In prokaryotes, these processes cannot be physically separated. Our data show that C-to-U editing separates termini trimming and intron removal and that *M. kandleri* tRNA maturation follows the order found in some eukaryotic tRNAs.

The purpose of the 30 C-to-U editing events in *M. kandleri* tRNAs is not known. It is plausible CDAT8 activity ensures that mature tRNAs contain the U8 base required for proper folding and function of the tRNA in protein biosynthesis. Therefore, the evolutionary advantage that resulted in the maintenance of C8 bases in the tRNA gene has to be determined by tRNA maturation stages before the editing event. Prevention of virus integration into tRNA genes (40) or the coordination of tRNA maturation events at extreme temperatures are possible scenarios. It was shown that U8 is usually found as a conserved modified 4-thiouridine nucleotide in bacterial and archaeal tRNAs, and the pathway for the biosynthesis of this modification was recently elucidated for methanogenic archaea (41). The presence of C8 in tRNA molecules would prevent introduction of this modification and might also coordinate local flexibility of the tRNA before formation of a U8:A14 tertiary base pair is possible, which stabilizes a sharp turn from acceptor stem to the D-stem of the tRNA. This entails that the timing of the occurrence of the U8 base during tRNA maturation in *M. kandleri* is important.

In conclusion, we describe the diverse sRNome for an organism that lives near the upper temperature limit for life and identified exceptionally high numbers of RNA modification guide RNAs and RNAs used for the defence against viruses. Both RNA families function even at extreme temperatures on the basis of annealing with their target nucleic acids. Increased RNA methylation and RNA circularization are suggested adaptations to the hyperthermophilic life style. Finally, the unique tRNA C-to-U event might be used to coordinate tRNA maturation.

## SUPPLEMENTARY DATA

Supplementary Data are available at NAR Online: Supplementary Table 1 and Supplementary Figures 1 and 2.

## FUNDING

Deutsche Forschungsgemeinschaft [DFG, FOR1680]; LOEWE Center for Synthetic Microbiology (Synmikro); the Max-Planck Society. Funding for open access charge: Max-Planck Society.

*Conflict of interest statement*. None declared.

## Supplementary Material

Supplementary Data
