# Supplementary material for: RNA-Seq analyses reveal the order of tRNA processing events and the maturation of C/D box and CRISPR RNAs in the hyperthermophile Methanopyrus kandleri
Source: Nucleic Acids Res. 2013 Apr 25;41(12):6250–8. doi: 10.1093/nar/gkt317 (PMC3695527; doi:10.1093/nar/gkt317)
Supplement: Supplementary Data [file supp_41_12_6250__index.html]

RNA-Seq analyses reveal the order of tRNA processing events and the maturation of C/D box and CRISPR RNAs in the hyperthermophile Methanopyrus kandleri — RNA-Seq analyses reveal the order of tRNA processing events and the maturation of C/D box and CRISPR RNAs in the hyperthermophile Methanopyrus kandleri — Supplementary Data 

# RNA-Seq analyses reveal the order of tRNA processing events and the maturation of C/D box and CRISPR RNAs in the hyperthermophile *Methanopyrus kandleri*

## Supplementary Data

files

**Files in this Data Supplement:**

- Supplementary Data - zip file
