# Supplementary material for: RNA-Seq analyses reveal the order of tRNA processing events and the maturation of C/D box and CRISPR RNAs in the hyperthermophile Methanopyrus kandleri
Source: Nucleic Acids Res. 2013 Apr 25;41(12):6250–8. doi: 10.1093/nar/gkt317 (PMC3695527; doi:10.1093/nar/gkt317)
Supplement: Supplementary Data [file supp_gkt317_nar-00764-v-2013-File007.zip › Su_Suppl_Material.pdf]

Supplemental Material

RNA-Seq analyses reveal the order of tRNA processing events and the maturation of C/D box and CRISPR RNAs in the hyperthermophile *Methanopyrus kandleri*

Andreas A. H. Su, Vanessa Tripp, Lennart Randau

Content:

Figures S1,S2

Legends for Table S1

Figure S1

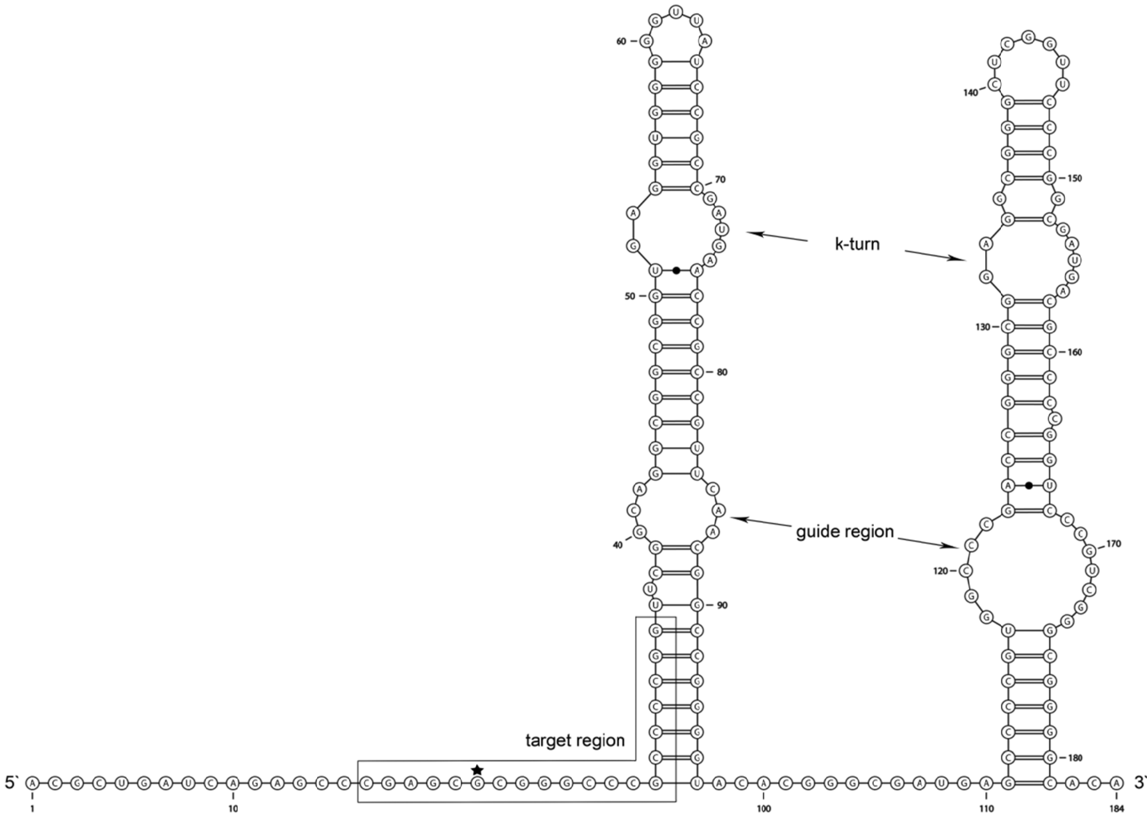

Figure S1

Proposed structure of H/ACA box sRNA 6 and potential methylation target region. Two kink-turn (k-turn) motifs and two pseudouridylation guide regions are indicated. Target prediction of C/D box sRNAs with the PLEXY tool (Kehr et al. 2011) identified a region for 2′-*O*-methylation (star) in this RNA. This region is targeted by C/D box sRNA 117 with the best overall quality score.

Figure S2

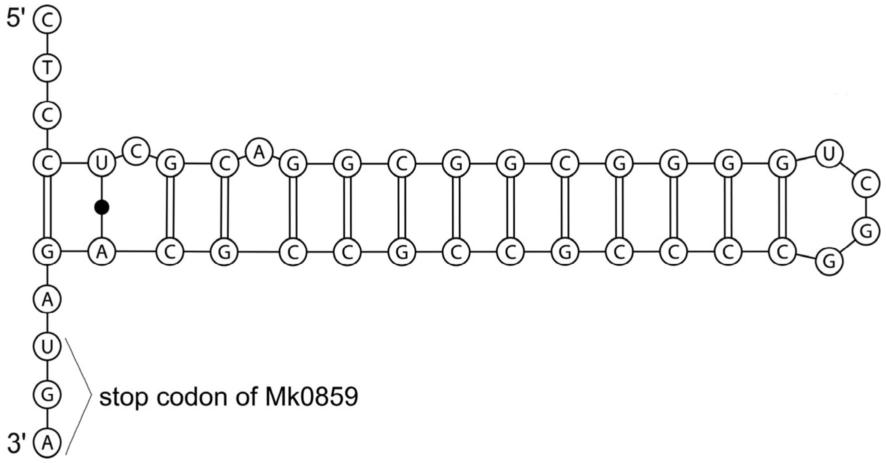

Figure S2

3′-terminal hairpin of a highly abundant mRNA. The high number of sequencing reads mapping to MK0859 (GAR1, member of the H/ACA ribonucleoprotein complex) correlate with an unusually stable hairpin directly upstream of the stop codon of the MK0859 open reading frame.

Table S1

Worksheet1

Transfer RNA and tRNA precursors. Transfer RNAs were identified and incorrect Genbank annotations were corrected (red sequences). 5′ leader regions and intron boundaries are indicated (modified from (Chan and Lowe 2009)). Mapped tRNA sequence reads for three independent RNA sample libraries without T4 PNK treatment are given and the tRNA reads of sample 1 were manually sorted to identify tRNA precursors with observed C8-to-U8 editing (C8U reads), leader and intron sequences.

Worksheet2

C/D box sRNA and potential targets. The table contains location, abundance and sequence of 126 identified C/D box sRNAs. C box motifs (consensus (GA)TGATGA) and C′ box motifs (consensus TGATGA) are indicated in red and D or D′ box motifs (consensus CNGA) are indicated in green. Potential terminal hairpins are underlined. Circularization was observed by the presence of permuted sequence reads (+) and occasionally the majority of reads were permuted (++). Target prediction of identified C/D box sRNAs was performed with the PLEXY tool (Kehr et al. 2011) against (i) a set of all tRNAs and rRNAs and (ii) a set of all identified RNA in this study. The position of the predicted 2′-*O*-methylation site and the PLEXY quality scores are indicated.

Worksheet3

CRISPR RNAs. The table lists identified crRNAs, their location and abundance. The 5′ terminal 8 nt tag and exonucleolytically degraded 3′ tags are underlined.

Worksheet4

List of identified sRNAs. This list covers sequence, location and abundance of all identified sRNAs that do not belong to the categories covered in Tables S1 – S3. Potential kink-turn motifs are indicated in red and ACA motifs are highlighted in blue.
